# Supplementary material for: Prophylactic protection against respiratory viruses conferred by a prototype live attenuated influenza virus vaccine
Source: Res Sq. 2021 Aug 13:rs.3.rs-668116. Preprint. [Version 1] doi: 10.21203/rs.3.rs-668116/v1 (PMC8366806; doi:10.21203/rs.3.rs-668116/v1)
Supplement: Supplement 1 [file a8ebdb5a4b29f03e0f69dc5b.pdf]

Supplementary Table 1. Units of IFN present in the allantoic fluid of 10-days embryonated chicken eggs which were inoculated with WT A/PR8/34 or ΔNS1 influenza A viruses.

**Virus**[a]	**Egg number**	**IFN (Uml^{-1})**[b]
**rWT PR8**	1	<16
	2	<16
**ΔNS1**	3	400
	4	400
**Mock**	5	<16
	6	<16

[a]Eggs were inoculated with 10^3 PFU of rWT-PR8 or ΔNS1 virus

[b]Amount of IFN in the allantoic fluid was measured 18 hours post inoculation

Supplementary Table 2. Viral titers, bodyweight changes and lung weights in A2G mice infected with ΔNS1 and hvPR8 viruses.

**Virus**	**Day 3**				**Day 6**			
	Mouse	PFU/ml[a]	Bodyweight changes [b]	Lung weight	Mouse	PFU/ml[a]	Bodyweight changes [b]	Lung weight
**ΔNS1**[c]	1	<10	+0.73 g	0.13 g	7	<10	+1.59 g	0.14 g
	2	<10	+1.19 g	0.13 g	8	<10	+2.89 g	0.15 g
**hvPR8**[e]	3	3×10^8	-3.05 g	0.32 g	9	3×10^6	-6.65 g	0.34 g
	4	3×10^7	-3.09 g	0.27 g	10	7×10^6	-4.69 g	0.24 g
**ΔNS1+ hvPR8**[d]	5	2×10^4	+1.58 g	0.15 g	11	<10	-0.03 g	0.12 g
	6	6×10^2	-0.42 g	0.11 g	12	1×10^4	+0.37 g	0.11 g

[a] Lungs were homogenized in 2 ml of PBS and viral titers were determined by standard plaque assay

[b] Numbers represent differences with respect to the bodyweight at the time of infection

[c] Animals were treated intranasally on day 0 with $2x10^5$ PFU of ΔNS1 virus

[d] Animals were treated intranasally on day -1 with $2x10^5$ PFU of ΔNS1 virus followed by intranasal challenge on day 0 with $2x10^4$ PFU of hvPR8 virus

[e] Animals were infected intranasally on day 0 with $2x10^4$ PFU of hvPR8
